# Circulating lymphocyte subsets as promising biomarkers to identify septic patients at higher risk of unfavorable outcome

**DOI:** 10.1186/s12879-021-06481-1

**Published:** 2021-08-09

**Authors:** Ennio Polilli, Jessica Elisabetta Esposito, Antonella Frattari, Francesca Trave, Federica Sozio, Giovanna Ferrandu, Giancarlo Di Iorio, Giustino Parruti

**Affiliations:** 1 Clinical Pathology Unit, Pescara General Hospital, Pescara, Italy; 2 Intensive Care Unit, Pescara General Hospital, Pescara, Italy; 3 Infectious Diseases Unit, Pescara General Hospital, Pescara, Italy; 4 Internal Medicine Unit, Pescara General Hospital, Pescara, Italy

**Keywords:** Sepsis, Clinical outcome, Lymphocytes, qSOFA, Lymphocyte subsets

## Abstract

**Background:**

Early recognition of patients hospitalized for sepsis at higher risk of poor clinical outcome is a mandatory task and many studies suggested that indicators of the immune status may be useful for this purpose. We performed a retrospective, monocentric cohort study to evaluate whether lymphocyte subsets may be useful in predicting in-hospital mortality of septic patients.

**Methods:**

Data of all consecutive patients with a diagnosis of sepsis at discharge and an available peripherical blood lymphocyte subset (CD4, CD8, CD16/CD56 and CD19) analysis at hospital entry were retrospectively collected between January 2015 and August 2018. Clinical characteristics of patients, past medical history and other laboratory parameters were also considered.

**Results:**

Two-hundred-seventy-eight septic patients, 171 (61.5%) males, mean age 63.2 ± 19.6 years, were enrolled. Total counts of lymphocytes, CD4 T cells, CD8 T cells and B cells were found significantly lower in deceased than in surviving patients. At univariate analyses, CD4 T cells/µL (OR 0.99 for each incremental unit, 95%CI 0.99–1.10, p < 0.0001), age (OR 1.06, 95%CI 1.04–1.09, p < 0.0001), procalcitonin (OR 1.01, 95%CI 1.01–1.02, p < 0.0001) and female gender (OR 2.81, 95%CI 1.49–5.28, p = 0.001) were associated with in-hospital mortality. When a dichotomic threshold of < 400/µL for CD4 T cells as a dependent variable was considered in multivariate models, age (OR 1.04; 95%CI 1.01–1.09, p = 0.018); female gender (OR 3.18; 95%CI 1.40–7.20, p = 0.006), qSOFA (OR 4.00, 95%CI 1.84–8.67, p < 0.001) and CD4 T cells < 400/µL (OR 5.3; 95%CI 1.65–17.00, p = 0.005) were the independent predictors.

**Conclusions:**

In adjunct to biomarkers routinely determined for the prediction of prognosis in sepsis, CD4 T lymphocytes, measured at hospital entry, may be useful in identifying patients at higher risk of in-hospital death.

**Supplementary Information:**

The online version contains supplementary material available at 10.1186/s12879-021-06481-1.

## Background

Sepsis has been reported as a major cause of increased morbidity, length of stay and mortality among hospitalized patients [1–4]. Improvement in survival remains contingent on early recognition, accurate stratification of risk factors for ICU transfer and on timely appropriate choice of therapies, including antibiotics [1, 4–6]. As a consequence, defining the individual risk of clinical progression after diagnosis of sepsis became a well-recognized priority [1, 5, 7, 8]. Unfortunately, this is complicated task, due to the highly variable and non-specific nature of symptoms and signs of ensuing sepsis [1, 5]. Clinical scores, such as qSOFA or NEWS2, alert clinicians to closely monitor patients at higher risk of organ dysfunction, but insufficient sensitivity was demonstrated in multiple settings, with the consequence of missed cases with occult hypoperfusion due to ensuing sepsis [3, 9]. Biomarkers such as C-reactive protein, Procalcitonin and Presepsin may have an important role in predicting patients’ prognosis, but all of them demonstrated insufficient sensitivity for this purpose in many settings of care [10–13].

Sepsis has been redefined as organ dysfunction caused by a dysregulated host response to infection [5]. Notably, lymphopenia and immune-suppression have been frequently described during the acute phase of infections by various pathogens, caused by pro-inflammatory cytokine response and possibly allowing progression of bacterial infections [14]. Persistent lymphopenia has been associated with unfavorable outcome in patients with sepsis, and many studies suggested that quantitative and qualitative characterization of lymphocyte subsets may be useful to refine prediction of sepsis outcome [15–20]. In the present study, we aimed to investigate the hypothesis that assay of circulating lymphocyte subsets at hospital entry may help predict in-hospital mortality in a large sample of patients hospitalized for sepsis at an Infectious Diseases Unit.

## Methods

We performed a retrospective, monocentric cohort study for the evaluation of lymphocyte subsets in the prediction of mortality of septic patients hospitalized at the Infectious Diseases Unit of Pescara General Hospital, Pescara, Italy. The study was conducted in accordance with the amended Declaration of Helsinki. As previously reported, the local Health Administrative Board reviewed in detail the study plan prepared by the Infectious Diseases Unit and Laboratory Staff of the Pescara General Hospital [21]. Written informed consent from all patients upon hospital admission was collected as previously described, and data used in this study was anonymised before its use [21].

Consecutive patients with diagnosis of sepsis at discharge and with available assays of lymphocyte subsets at hospital entry were enrolled between January 2015 and August 2018. Lymphocyte subsets were assayed using flow cytometry and percentages of CD4 T lymphocytes (T-helper cells), CD8 T cells (Cytotoxic T cells), CD16/CD56 Natural Killer cells and CD19 B-lymphocytes were evaluated by the Aquios CL Instrument (Backman Coulter, Inc). Clinical characteristics of patients, including age, sex, vital signs, past medical history and other laboratory data were collected upon enrolment as described in Shao et al. (2015) [22]. Diagnosis of sepsis or septic shock were defined according with the criteria of the Sepsis 2 (2002) [23], associated with the ICD-9-CM sepsis codes at hospital discharge. Criteria for organ dysfunction were applied as described in Shao et al. (2015) [22].

Epidemiological and clinical factors analyzed were age; gender; qSOFA score; complete blood count; renal function; hepatic function; C-Reactive Protein (CRP) and procalcitonin (PCT). RBC (Red Blood Cells); HGB (Hemoglobin); HCT (Hematocrit); MCV (Mean Corpuscular Volume); MCH (Mean corpuscular hemoglobin); MCHC (Mean Cell Hemoglobin Concentration); MPV (Mean Platelet Volume); PDW (Platelet Cell Width), RDW (Red Cell Distribution Width) and platelet indices were performed with the Unicell DX 800 Instrument (Beckman Coulter, Brea, California). Blood cultures drawn upon suspicion of sepsis were processed in the local Microbiology Unit. The burden of comorbidities was evaluated by the Charlson Comorbidity Index, calculated as described by Charlson et al. [24]. Antibiotic prescribed and days of hospitalization were also considered. Previous antibiotic exposure was defined as at least one week of either quinolone, beta-lactam or carbapenem prescribed in the month preceding hospitalization.

Adverse outcomes were defined as sepsis relapse, if a second episode of sepsis occurred within 3 months from discharge, and death during hospitalization. Enrolled patients who died from any cause during hospitalization were classified as non-survivors.

Differences in the selected variables were analyzed as previously described [21]. Logistic regressions were used to assess the independent association between death and each included variable. Statistical significance was defined as a two-sided p-value < 0.05, as previously described [21].

## Results

We enrolled 278 septic patients, 171 (61.5%) males, mean age 63.25 ± 19.65 years. In 21.4% of patients qSOFA was 1; in 51.1% 2 and in 2.2% 3. Described in Table 1 are the frequencies of comorbid conditions: a CCI ≤ 3 was present in 39.9% of patients; a CCI of 4–6 in 36.7% of patients and a CCI > 6 in the remaining 23.4%. Mean of hospitalization was 16.6 ± 11.6 days; relapse of sepsis was observed in 43 (15.4%) patients while 49 (17.6%) patients died during their hospital stay. Blood cultures were positive in 137 (49.3%) of patients during any time of stay in the ward; in 108 patients these yielded 1 microorganism; in 20 2 microorganisms, whereas > 2 microorganisms were retrieved in the remaining 9 patients. Features of survivors and non-survivors, including the distribution of potential predictors, are summarized in Table 1. The source of sepsis more frequently found were pneumonia (91 patients, 32.7%), gastrointestinal infections (50 patients, 18.0%) and urinary tract infections (39 patients, 14.0%).

**Table 1:** Characteristics of patients associated with in-hospital mortality

Variables	Overall 278 (100%)	Survivors 229 (82.4%)	Non survivors 49 (17.6%)	*p*
Age, *years, mean (SD)*	63.25 (19.65)	60.44 (19.81)	76.41 (12.28)	< 0.001
Males/females, *n (%)*	171/107 (61.5/38.5)	151/78 (65.9/34.1)	20/29 (40.8/59.2)	0.001
Charlson Comorbidity Index				
≤ 3, (%)	39.93	46.29	10.20	
4–6, (%)	36.69	33.19	53.06	
> 6, (%)	23.38	20.52	36.73	
*Mean (SD)*	4.23 (2.95)	3.85 (2.93)	6.0 (2.35)	< 0.001
Comorbid condition				
Myocardial infarction history	5 (1.8)	4 (1.7)	1 (2.0)	
Peripheral disease	14 (5.0)	10 (4.4)	4 (8.2)	
Cerebro-vascular disease	110 (39.6)	88 (38.4)	22 (44.9)	
Dementia	23 (8.3)	9 (3.9)	14 (28.6)	
Chronic pulmonary disease	62 (22.3)	52 (22.7)	10 (20.4)	
Connective tissue disease	6 (2.2)	4 (1.7)	2 (4.1)	
Peptic ulcer disease	3 (1.1)	1 (0.4)	2 (4.1)	
Mild liver disease	27 (9.7)	24 (10.5)	3 (6.1)	
Diabetes without end-organ damage	63 (22.7)	52 (22.7)	11 (22.4)	
Hemiplegia	5 (1.8)	3 (1.3)	2 (4.1)	
Moderate or severe renal disease	49 (17.6)	38 (16.6)	11 (22.4)	
Diabetes with end-organ damage	5 (1.8)	4 (1.7)	1 (2.0)	
Tumor without metastasis	26 (9.4)	17 (7.4)	9 (18.4)	
Leukemia	3 (1.1)	1 (0.4)	2 (4.1)	
Lymphoma	3 (1.1)	1 (0.4)	2 (4.1)	
Moderate or severe liver disease	6 (2.2)	6 (2.6)	0 (0.0)	
Metastatic solid tumor	3 (1.1)	2 (0.9)	1 (2.0)	
qSOFA, *mean (SD)*	1.30 (0.87)	1.16 (0.87)	1.96 (0.55)	< 0.001
PCT, ng/dL, *mean (SD)*	20.7 (31.1)	17.4 (28.1)	36.5 (39.5)	< 0.001
Positive blood culture, *n (%)*	137 (49.28)	113 (49.34)	24 (49.0)	0.96
Length of stay, *mean (SD)*	16.6 (11.6)	15.1 (10.0)	16.8 (11.9)	0.4
Infection				
Pneumonia	91 (32.7)	70 (30.6)	21 (42.9)	
Gastrointestinal infection	50 (18.0)	40 (17.5)	10 (20.4)	
Urinary tract infection (UTI)	39 (14.0)	35 (15.3)	4 (8.2)	
Skin and soft tissue infection	25 (9.0)	25 (10.9)	0 ( 0.0)	
Endocarditis	13 (4.7)	12 (6.3)	1 (2.0)	
Central venous catheter infection	4 (1.4)	4 (1.7)	0 (0.0)	
Pleuritis	4 (1.4)	2 (0.9)	2 (4.1)	
Peripherally inserted central catheter infection	3 (1.1)	2 (0.9)	1 (2.0)	
Wound infection	3 (1.1)	2 (0.9)	1 (2.0)	
Abscess	2 (0.7)	2 (0.9)	0 (0.0)	
Meningoencephalitis	2 (0.7)	2 (0.9)	0 (0.0)	
Osteomyelitis	2 (0.7)	2 (0.9)	0 (0.0)	
Unknown	40 (14.4)	31 (13.5)	9 (18.4)	

In our study, in-hospital mortality was tightly linked with age (76.41 ± 12.28 years vs 60.44 ± 19.81 years, p < 0.001); female gender (59.2% vs 34.1%, p = 0.001); CCI (6.0 ± 2.35 vs 3.85 ± 2.93, p < 0.001); qSOFA at hospital entry (1.96 ± 0.55 vs 1.16 ± 0.87, p < 0.001) and PCT (36.5 ± 39.5 vs 17.4 ± 28.1 ng/dL, p < 0.001). All other potentially relevant variables, such as positive blood cultures (49.0% vs 49.3%, p = 0.96) and length of stay (16.8 ± 11.9d vs 15.1 ± 10.0d, p = 0.4) failed to reveal any association (Table 1).

Among hematological variables, lymphocytes (0.6*10^3^/µL, [IQR 0.3–1.1] vs 1.1*10^3^/µL, [IQR 0.6–1.6], p < 0.001) as well as monocytes (0.5*10^3^/µL, [IQR 0.2–0.9] vs 0.7*10^3^/µL, [IQR 0.4–0.9], p = 0.002) and platelets (154*10^3^/µL, [IQR 74–229] vs 192*10^3^/µL, [IQR 138–262], p = 0.003) turned out to be associated with in-hospital mortality. Similarly, median CD3 T cells (347/µL, [IQR 188–536] vs 673/µL, [IQR 368–1,080], p < 0.001), median CD4 T cells (197/µL, [IQR 106–265] vs 405/µL, [IQR 222–661], p < 0.001), median CD8 T cells (134/µL, [IQR 87–231] vs 268/µL, [IQR 133–461], p < 0.001) and median CD19 B cells (76/µL, [IQR 35–140] vs 130/µL, [IQR 67–227], p < 0.001), that is lymphocyte subsets possibly linked with mortality in sepsis, based on evidence in other settings, revealed significant association, as shown in Table 2. Median WBC (12.9*10^3^/µL, [IQR 6.8–17.1] vs 11*10^3^/µL, [IQR 7.2–15.2], p = 0.53) and Neutrophils (10.6*10^3^/µL, [IQR 6.4–15.5] vs 8.7*10^3^/µL, [IQR 4.9–13], p = 0.2) failed to reveal any association (Table 2).

**Table 2:** Hematological parameters associated with in-hospital mortality

Variable	Survivors n = 229	Non-survivors n = 49	*p*^§^
	Median (IQR)	Median (IQR)	
WBC, *10^3^/µL	11 (7.2–15.2)	12.9 (6.8–17.1)	0.53
Neutrophils, *10^3^/µL	8.7 (4.9–13)	10.6 (6.4–15.5)	0.20
Lymphocytes, *10^3^/µL	1.1 (0.6–1.6)	0.60 (0.3–1.1)	< 0.001
Monocytes, *10^3^/µL	0.7 (0.4–0.9)	0.5 (0.2–0.9)	0.002
Platelet, *10^3^/µL	192 (138–262)	154 (74–229)	0.003
CD3 + T Lymphocytes, cells/µL	673 (368–1,080)	347 (188–536)	< 0.001
CD4 + T Lymphocytes, cells/µL	405 (222–661)	197 (106–265)	< 0.001
CD8 + T Lymphocytes, cells/µL	268 (133–461)	134 (87–231)	< 0.001
CD16/56 + Lymphocytes, cells/µL	34 (14–91)	37 (16–106)	0.66
CD19 + B Lymphocytes, cells/µL	130 (67–227)	76 (35–140)	< 0.001

*WBC* white blood cell count, *IQR* interquartile range

^§^Calculated with Kruskal–Wallis

We calculated sensitivity and specificity as well as PPV and NPV of different CD4 T cells cut points for the prediction of in-hospital mortality. Best sensitivity and specificity for CD4 T lymphocytes were at values < 400/µL (87.8%, IQR 75.2–95.4) and < 200/µL (77.7%, IQR 71.8–82.9), respectively (Table 3). At univariate analyses, CD4 T cells/µL (OR 0.99, 95%CI 0.99–1.10, p < 0.0001), age (OR 1.06, 95%CI 1.04–1.09, p < 0.0001), procalcitonin (OR 1.01, 95%CI 1.01–1.02, p < 0.0001) and female gender (OR 2.81, 95%CI 1.49–5.28, p = 0.001) were associated with death (Table 4).

**Table 3:** Sensitivity and specificity of different cut-points of lymphocytes T-CD4 in predicting in-hospital mortality

Variable	Sensitivity, % (IQR)	Specificity, % (IQR)	PPV, % (IQR)	NPV, % (IQR)
CD4 + T cells < 200/µL	55.1 (40.2–69.3)	77.7 (71.8–82.9)	35.6 (24.2–46.2)	89.9 (83.8–93.0)
CD4 + T cells < 250/µL	67.3 (52.5–80.1)	70.7 (64.4–76.5)	33.0 (23.9–43.1)	91.0 (85.8–94.8)
CD4 + T cells < 300/µL	81.6 (68.0–91.2)	64.2 (57.6–70.4)	32.8 (24.6–41.9)	94.2 (89.3–97.3)
CD4 + T cells < 350/µL	85.7 (72.8–94.1)	59.4 (52.7–65.8)	31.1 (23.4–39.6)	95.1 (90.2–98.0)
CD4 + T cells < 400/µL	87.8 (75.2–95.4)	52.0 (45.3–58.6)	28.1 (21.1–35.9)	95.2 (89.8–98.2)
CD4 + T cells < 450/µL	87.8 (75.2–95.4)	43.2 (36.7–49.9)	24.9 (18.6–32.0)	94.3 (88.0–97.9)

*PPV* positive predictive value, *NPV* negative predictive value

**Table 4:** Logistic regression models predicting in-hospital mortality with continuous and categorical values of CD4 + T cells

Variable	Crude OR (95%CI)**	Univariate p	Adjusted OR (95%CI)**	Multivariate p	Adjusted OR (95%CI)^##^	Multivariate p
Age (1 year increase)	1.06 (1.04–1.09)	< 0.0001	1.04 (1.01–1.09)	0.021	1.04 (1.01–1.09)	0.018
Female gender	2.81 (1.49–5.28)	0.001	3.35 (1.54–7.99)	0.003	3.18 (1.40–7.20)	0.006
CCI*	1.30 (1.15–1.45)	< 0.0001	1.10 (0.91–1.33)	0.321	1.09 (0.90–1.32)	0.373
qSOFA*	5.12 (2.60–10.08)	< 0.0001	3.85 (1.77–8.41)	0.001	4.00 (1.84–8.67)	< 0.001
PCT, ng/mL*	1.01 (1.01–1.02)	< 0.0001	1.01 (1.00–1.02)	0.198	1.01 (1.00–1.02)	0.227
CD4 + T, cells/µL*	0.99 (0.99–1.10)	< 0.0001	0.99 (0.99–1.00)	0.002	–	–
CD4 + T, cells < 400/µL	7.75 (3.18–18.93)	< 0.0001	–	–	5.3 (1.65–17.00)	0.005
PLT/µL*	0.99 (0.99–1.00)	0.005	1.00 (1.00–1.01)	0.409	1.01 (1.00–1.01)	0.374
Lymphocytes, cells/µL*	0.32 (0.17–0.58)	< 0.0001	0.97 (0.58–1.64)	0.919	0.70 (0.31–1.58)	0.398

*CCI* Charlson Comorbidity Index, *qSOFA* quick Sequential Organ Failure Assessment, *PCT* procalcitonin, *PLT* platelet, *OR* odds ratio, *IC* confidence interval;

*1-unit increase

Parameters of the models: ** Hosmer–Lemeshow goodness of fit p = 1.00; area under the receiving operator curve (ROC) = 0.89;

^##^Hosmer–Lemeshow goodness of fit p = 0.99; area under the receiving operator curve (ROC) = 0.89

In multivariate analyses, the variables independently associated with in-hospital mortality were age (OR 1.04 for each incremental year; 95%CI 1.01–1.09, p = 0.021); female gender (OR 3.35; 95%CI 1.54–7.99, p = 0.003), qSOFA (OR 3.85 for each incremental unit; 95%CI 1.77–8.41, p = 0.001) and CD4 T lymphocytes (OR 0.99 for each incremental unit; 95%CI 0.99–1.00, p = 0.002). No association was found for CCI (OR 1.10; 95%CI 0.91–1.33, p = 0.321); PCT (OR 1.01; 95%CI 1.00–1.02, p = 0.198); lymphocytes (OR 0.97; 95%CI 0.58–1.64, p = 0.919) and PLT (OR 1.00; 95%CI 1.00–1.01, p = 0.409), as shown in Table 4. When CD4 T lymphocytes were dichotomized at < 400/µL in a new model, age (OR 1.04 for each incremental year; 95%CI 1.01–1.09, p = 0.018); female gender (OR 3.18; 95%CI 1.40–7.20, p = 0.006), qSOFA (OR 4.00 for each incremental unit; 95%CI 1.84–8.67, p < 0.001) and CD4 T cells < 400/µL (OR 5.3; 95%CI 1.65–17.00, p = 0.005) were the significant independent predictors of mortality. CCI (OR 1.09; 95%CI 0.90–1.32, p = 0.373); PCT (OR 1.01; 95%CI 1.00–1.02, p = 0.227); lymphocytes (OR 0.70; 95%CI 0.31–1.58, p = 0.398) and PLT (OR 1.01; 95%CI 1.00–1.01, p = 0.374, Table 4) once more failed to reveal any significant association.

## Discussion

For years SIRS criteria (SEPSIS 2) have been considered the best screening tool to recognize septic patients. However, SEPSIS 2 criteria were criticized across the years, because of inadequate specificity and sensitivity [5, 25–27]. As a consequence, in 2016 the European Society of Intensive Care Medicine and the Society of Critical Care Medicine task force delivered a Third International Consensus definition for sepsis, to replace screening criteria of SIRS with the qSOFA score, that was validated to pick up patients with poor outcomes out-of-hospital, in the emergency department, and in general hospital wards (SEPSIS 3) [5]. To date, several studies examined the predictive performance of qSOFA for in hospital mortality, revealing low sensitivity at the time of initial suspicion of infection [28]. Furthermore, several clinical scores were evaluated for their ability to recognize patients at higher risk of death, but a recent systematic review and meta-analysis suggested that Early Warning Scores and qSOFA not always predict mortality accurately in patients with sepsis [29, 30]. In addition, many studies in the literature tested the independent value of biochemical markers for their ability to predict sepsis, without conclusive results, leaving clinicians in the need of further aid [31–34]. The evaluation of specific subsets of peripheral immune cells may, indeed, improve current prediction models to timely recognize septic patients at higher risk of poor outcome, taking into account the immunological status at onset of sepsis [27].

In our pivotal study, we found that an absolute number of < 400/µL CD4 T lymphocytes at hospital entry was independently and strongly associated with in-hospital mortality in a large sample of patients hospitalized at an Infectious Disease Unit with the suspect of sepsis. To complement the relevance of our finding, having CD4 T lymphocytes > 400/µL on admission had a very high NPV (95.2%) for in hospital mortality, once more suggesting that this immune parameter may be useful for an early prediction of prognosis in patients with sepsis.

Lymphocytes are an essential part of the adaptive immune response to infection and their subsets play a well-documented and complex role in sepsis [17]. A drop in the absolute number of total T-lymphocytes was observed upon hospital admission in patients with infection, but the role of subsets of lymphocytes in predicting survival and non-survival of patients with sepsis has been so far poorly investigated [16]. Profound and persistent lymphopenia, partly reflecting migration of T-cells into infection sites, is closely related to infection and sepsis, being associated with expansion of immunosuppressive cell populations such as regulatory T-lymphocytes, IL-10-producing B-lymphocytes and myeloid-derived suppressor cells, whose rise may last for months [18–20]. Immune homeostasis is perturbed by a strong inflammatory response, as in a septic episode, followed by a rapid negative feedback due to the compensatory anti-inflammatory response, leading a decrease in functional T-lymphocytes [19, 35, 36]. Previous studies indicated that lymphopenia may occur early in the course of sepsis and that decreases of specific lymphocyte subsets, including CD4 T cells, may help identify fragile patients at higher risk of disease progression among those hospitalized due to infection [18–20]. As a consequence, qualitative and quantitative changes of different immune cell subsets were investigated in sepsis, aimed to the prediction of clinical progression in septic patients [16, 37]. These studies, however, commonly enrolled critically ill ICU patients, and investigated small samples of patients [16, 37]. Wu et al. (2013) enrolled 87 ICU patients with severe sepsis, showing higher numbers of CD4 T-Th1 lymphocytes in survivors [38]. Similarly, Hohlstein et al. (2019) demonstrated that numbers of T and NK lymphocyte at ICU admission were significantly higher in septic shock survivors than in non-survivors [35]. CD4 T lymphocytes play a pivotal role in response to microbial spread [18] and decreased circulating CD4 T lymphocytes may increase the risk of acquiring bloodstream infections [20]. In critically ill patients dying with sepsis-related multi-organ dysfunction, Boomer et al. (2011) demonstrated that lymphocyte dysfunction was found both in peripheral blood, and in lymphoid organs [39]. In this frame of current knowledge, our study is the first to enroll a large and consecutive sample of non-critical, relatively stable patients hospitalized in an Infectious Diseases ward due to the suspect of ensuing sepsis; indeed, only a handful of such patients were hospitalized with septic shock, in line with current recommendations. Among the numerous immune subsets measured, CD4 T lymphocytes were tightly associated with prognosis when assayed upon hospitalization, thus providing further evidence that CD4 T cell deficits and/or dysfunction may occur early in the process of dysregulated immune response to bacterial spread, being likely associated both to the pathogenesis and to the prognosis of sepsis [18]. Our study therefore provides an additional line of evidence that analyzing lymphocyte subsets early in the evolution of sepsis may be considered a valuable tool in the establishment of an immune prognostic score for sepsis. Interestingly, the tight and strong association of CD4 T lymphocytes with sepsis prognosis was quite relevant in comparison with other biochemical markers commonly used to stratify outcome of patients with sepsis [36].

Our study design has a few limitations. First, it was a single center study, with prospective enrollment and retrospective evaluation of the results. Immune characterization was prescribed by attending physicians in the ward to most, but not to all patients hospitalized with the suspicion of sepsis, and this may have caused some enrollment bias. Second, non-survivors in our cohort, that is patients dying due to sepsis among those enrolled, were relatively few, which made it difficult to control for confounding factors and possible selection biases in our multivariate analyses. Third, we one more acknowledge the monocentric nature of our study design and a multicentric study is needed to confirm our preliminary data. Four, we did not perform any sequential analysis of lymphocyte subsets in the weeks following hospitalization, which might have been useful to monitor possible lymphocyte subset variations related to hospital treatments. As a consequence, a prospective, multi-center study with planned enrollment of a larger sample of patients, including a second assay of lymphocyte subsets after 2–4-weeks, will be necessary to further validate our preliminary evidence that CD4 T-cells measurements may help predict mortality in septic patients.

## Conclusions

Our results suggest that lymphocyte subsets, and in particular CD4 T lymphocytes, measured at hospital entry in patients with ensuing sepsis, may be useful to identify those at higher risk of death during their stay in medical wards, complementing biochemical markers and other scores routinely used to this purpose. Further research is warranted (Additional file 1).

## Supplementary Information

**Additional file 1.** Database file for analyses.

## Data Availability

The datasets analyzed during the current study are available from the corresponding author on reasonable request. No administrative permissions will be required to access the raw data mentioned in the methods upon request.

## Abbreviations

CCI: Charlson Comorbidity Index
IQR: Interquartile range
NPV: Negative predictive value
OR: Odds ratio
PCT: Procalcitonin
PLT: Platelet
PPV: Positive predictive value
qSOFA: quick Sequential Organ Failure Assessment
WBC: White blood cell count
